# The Expression of *TLR2* and *TLR3* in Sertoli Cells of Azoospermic Patients 

**DOI:** 10.22074/cellj.2017.4300

**Published:** 2017-08-19

**Authors:** Mohammad Reza Lakpour, Morteza Koruji, Abdolhossein Shahverdi, Samaneh Aghajanpour, Majid Rajabian Naghandar, Mohammad Ali Sadighi Gilani, Marjan Sabbaghian, Reza Aflatoonian

**Affiliations:** 1 Department of Andrology, Reproductive Biomedicine Research Center, Royan Institute for Reproductive Biomedicine, ACECR, Tehran, Iran; 2 Department of Biology, Payam Noor University, Tehran, Iran; 3 Cellular and Molecular Research Center, Department of Anatomical Sciences, Iran University of Medical Sciences, Tehran, Iran; 4 Department of Embryology, Reproductive Biomedicine Research Center, Royan Institute for Reproductive Biomedicine, ACECR, Tehran, Iran; 5 Department of Endocrinology and Female Infertility, Reproductive Biomedicine Research Center, Royan Institute for Reproductive Biomedicine, ACECR, Tehran, Iran; 6 Department of Urology, Shariati Hospital, Tehran University of Medical Sciences, Tehran, Iran

**Keywords:** Sertoli Cells, Testis, Fibroblast Cells, *TLRs*

## Abstract

**Objective:**

Toll-like receptors (*TLRs*) on Sertoli cells are thought to have essential roles in sperm protection. This study was conducted to investigate the expression of *TLR2* and *TLR3* in Sertoli cells of men with azoospermia.

**Materials and Methods:**

In this experimental study, testicular biopsies were taken from ten azoospermic men. Following enzymatic dissociation, the samples were moved to lectin coated petri dishes. After a few passages, all cells were cultivated and Seroli cells were sorted by flow cytometry. To confirm Sertoli cell purification, alkaline phosphatase activity (ALP) and immunohistochemistry assays were employed. The expression of *TLR2* and *TLR3* at the transcript and protein levels was examined with real-time quantitative reverse transcription-polymerase chain reaction (RT-QPCR) and western blot, respectively.

**Results:**

Isolation, purification and cultivation of human Sertoli cells were performed successfully. Efficacy of purification of Sertoli cells by fluorescence-activated cell sorting (FACS) sorter was ~97%. The type of cultured cells was confirmed by vimentin and follicle-stimulating hormone (FSH) receptor markers. Furthermore, the existence of anti- Müllerian hormone in culture was confirmed. RT-PCR showed that both genes were expressed in Sertoli cells. Consistently, proteins of both were also expressed in Sertoli cells. Moreover, QPCR showed that the relative expression of *TLR3* transcripts was significantly higher than *TLR2* in Sertoli cells. Although both genes are expressed in fibroblast cells, their level of expression was significantly lower than in Sertoli cells.

**Conclusion:**

This study confirmed expression of *TLR2* and *TLR3* in human Sertoli cells. This may be an indicator of their roles in developing immunity against pathogens as well as allo- and auto-antigens or viral antigens in seminiferous tubules.

## Introduction

Sertoli cells are a type of testicular cells located on the basal membrane of seminiferous tubules; their cytoplasm has developed toward the lumen and surrounds various types of proliferating and differentiating germ cells (1). They have an important function in the nourishment of germ cells through all stages of spermatogenesis (2). Additionally, they have a main role in phagocytosis of excess spermatid cytoplasm during spermatozoa formation (3) and also secrete some hormones like anti-Müllerian and inhibin (4). Toll-like receptors (*TLRs*) are one of the main groups of pathogen associated molecular patterns (PAMPs). These recognition pattern receptors recognize molecular patterns of pathogens and aid the innate immune system in detecting invading pathogens (5). Different *TLRs* respond to different associated molecular patterns with pathogens including lipopolysaccharide (*TLR4*), lipopeptides (*TLR1, 2, 6*), bacterial flagella (*TLR5*), double strand RNA virus (*TLR3, 7, 8*) and un-methylated DNA rich in CpG (*TLR9*) (6). *TLR*, as a mediator, not only has a vital function in activating innate immunity, but also is a bridge between innate and adaptive immunity. Also, *TLRs* are expressed in both immune and non-immune cells including B lymphocytes, natural killer (NK) cells, dendritic cells, macrophages, fibroblasts, epithelial and endothelial cells (7,8). Additionally, these receptors have the capability of dimerization on the surface of the cell membrane, in which either two identical proteins are hemodimerised or two different *TLRs* are heterodimerised. Heterodimerisation has been shown to increase specificity of these receptors (9). 

Among the *10 TLR* members in humans, *TLR1, 2, 4, 5* and *6* were shown to be located on the surface of the cells and attached to molecular patterns of extracellular microbes, however, *TLR3, 7, 8* and *9* were expressed on the membrane of cytoplasmic organelles particularly endosomes to detect nucleic acids associated with pathogens (10). It was also revealed that all *TLRs* except *TLR10* are present on the sperm and proved *TLR4* amounts high expressed in sperm, showing the importance of this receptor in generating a safe environment in testis (11). Presence of these receptors on dermal fibroblasts was investigated recently, which shows all *TLRs* are expressed on fibroblasts (12). Sertoli cells display differential cytokine responses to bacterial stimuli from those of testicular macrophages, which are mediated by both *TLR2* and *TLR4* (13). 

Based on previous studies, expression of *TLR1- 10* transcripts in animals tissues of the testis has been demonstrated (14). In addition, it has been shown that not only rat testis but also epididymis and vas deferens express *TLR1-9* transcripts (15). Although *TLRs* activate the immune system and induce immune responses against pathogens, some of them may also cause auto-immune and acute inflammatory disorders such as cardiomyopathy which is one of the common reasons of cardiac failure in youth (16). Therefore studying the functional mechanisms of *TLRs* on Sertoli cells may aid in controlling inflammatory diseases of testicles (17). 

While Sertoli cells of the testis have a vital role in feeding and supporting the developing spermatozoids, no study has been undertaken in analyzing the expression of *TLR2* and *TLR3* at the transcript and protein levels in human Sertoli cells. By demonstrating the presence of *TLRs* on human Sertoli cells, it is possible to clarify the role of these cells in the immune system as local defense against pathogens. We therefore analyzed the expression of both genes at the transcript and protein levels in Sertoli cells of human testicular tissue. 

## Materials and Methods

In this experimental study, conducted to isolate and cultivate Sertoli cells, human samples were collected from ten obstructive azoospermic patients. The Ethical Committee of Royan Institute approved this study. These patients were admitted to Royan Institute, of whom all underwent surgery for testicular sperm extraction (TESE). Patients were informed of the procedure and all signed written consent before TESE. Each recruited patient had a complete medical history. 

### Isolation of testicular cells

Testicular cells were isolated according to previous studies with some modifications (18,19). For isolation of cells, at least 2-4 biopsies (3-5 mm) were taken from the testis of azoospermic patients. The samples were rinsed with phosphate-buffered saline (PBS, Sigma, USA) containing penicillin-streptomycin and gentamycin and were then placed in Dulbecco’s Modified Eagle’s Medium (DMEM, Gibco, USA). Next, testicular tissue samples were mechanically fragmented into small pieces in a medium culture containing digestive enzymes, namely collagenase (1 mg/ml, Sigma, St. Louis, MO, USA), Hyalorunidase (1500 IU, 100 µl/ ml) and DNase (1 mg/ml, Sigma, St. Louis, MO, USA), Trypsin (1 mg/ml, Sigma, St. Louis, MO, USA), and were then incubated for 30 minutes at 37˚C and mixed every 10 minutes by a sampler for 1 minute. Afterwards, the medium containing the cells and pieces of the seminiferous tubules were centrifuged several times until the supernatant became clear (3-4 times at 1100 rpm for 1 minute). After every centrifuge, the supernatant was then substituted by fresh DMEM. This was undertaken to remove interstitium, sperms and spermatids from the seminiferous tubules. 

### Enrichment and cultivation of Sertoli cells

For separation of the Sertoli cells, the resulting cells from enzymatic digestion were used to implement methods of studying Scarpino et al. (18) and the differentiation plating method of van Pelt (20). In summary, first, 5 μg /ml of lectin datura stramonium agglutinin (DSA, Sigma, USA) was solved in buffer phosphate and incubated at 37˚C for 1 hour. The petri dish was washed by phosphate-buffer containing 0.5% of bovine serum albumin (BSA, Sigma, USA). After drying, cellular solution was transferred to a petri dish covered by DSA lectin and incubated in 5% CO_2_ for 1 hour at 32˚C. After incubation, free cells were removed from the solution. Cells attached to the petri dish were somatic cells including Sertoli cells, which were then cultivated by adding DMEM containing 10% fetal bovine serum (FBS, Gibco, UK) for 3-4 days. Later, for separation of cells from the petri dish, they were treated with ethylenediamine tetraacetic acid- trypsin (EDTAtrypsin) in PBS with no Ca^2+^ and Mg^2+^ (Sigma, USA) for 5 minutes at 37˚C. The cells were again cultivated with cultivating medium containing 10% serum. This method separated Sertoli and myoid cells. Viability and cell count were determined by trypan blue staining. To improve the cultivation conditions of the Sertoli cells, 7000 IU/mg of human follicle-stimulating hormone (FSH, Sigma, USA) was added to cell cultivation medium.

### Flow cytometry

The Sertoli cells were detached from culture dishes by EDTA-trypsin and then washed with PBS and 2% FBS. For detection of FSH expression on Sertoli cell surface, anti-FSH receptor antibody (rabbit polyclonal antibody to FSH receptor, Abcam, USA) was used. To separate the two groups of cells (control and test groups); Sertoli cells needed to be separated by FACS. Just the primary antibody was added to cells in the test group and secondary antibody was added to both groups. For this, 20 μl of primary antibody FSH receptor (FSHr, 50 μg at 1 mg/ml, Abcam, USA) was added to 1×10^6^ cells in 100 μl PBS as the test group. After 45 minutes of incubation at 4˚C, cells were rinsed with 1 ml of PBS and 2% FBS solution and centrifuged (1500 rpm, 5 minutes at 4˚C), and the cell sediment was then treated with 300 μl of PBS. Subsequently, 3 μl of secondary antibody (goat polyclonal antibody to rabbit IgG FITC, Abcam, USA) diluted at 1:200 concentration was added to the test and control groups. Both samples were incubated at 4˚C for 45 minutes, washed with 1 ml of PBS and 2% FBS solution, and centrifuged at 1500 rpm for 5 minutes at 4˚C. The supernatant was removed and the sediment was then suspended in 1 ml of solution [PBS, EDTA, hydroxylethylpiperazine ethanesulfonic acid (HEPES) buffer, BSA 1%]. Finally, Sertoli cells sorted on the basis of FSHr expression were separated from other cells (mesenchymal fibroblasts) by BD FACS aria II cell sorter (BD Biosciences).

### Immunocytochemistry

Cultured Sertoli cells on chamber slides were evaluated according to two markers, namely vimentin and FSH receptors. A group of cells were treated with anti-vimentin antibody and others with anti-FSH receptor antibody. These cells were fixed with 4% formaldehyde for 2 minutes. The cells were then treated with 0.3% Triton X-100 in PBS for 15 minutes at room temperature to increase cellular permeability. After blocking, cells were incubated with 10% normal goat serum in PBS at room temperature for 30 minutes. A group of cells were treated with mouse FITC-conjugated monoclonal anti-vimentin antibody (Abcam, USA) diluted at 1:50 concentration, while others were treated with rabbit polyclonal anti-FSH receptor antibody (Abcam, USA) diluted at 1:200 concentration for 24 hours at 4˚C. After three washes with PBS, the cells were incubated with the secondary antibody (goat FITC-conjugated anti Rabbit IgG, Abcam, USA) with 1:100 concentrations. The cells were finally mounted with a mounting medium (Vector Laboratories Inc., Burlingame, CA) after three washes with PBS and examined under a fluorescence microscope (IX-71, Olympus).

### Activity of alkaline phosphatase

To examine alkaline phosphatase (ALP) activity, chamber slides containing Sertoli cell were stained on the basis of a related ALP kit (Sigma, USA). Briefly, cells were fixed for 10 minutes in solution containing ethanol and acetone (1:1), followed by incubation for 30 minutes in Fast Blue RR solution (0.5 mg/ml, Sigma, USA) and α-naphtol phosphate 0.25% (Sigma, USA). After rinsing in water, the samples were mounted on an aqua- mount (BDH, Italy) and examined under optical microscopy. Mouse testicular tissue was used as a positive control. 

### Enzyme-linked immunosorbent assay

The concentration of anti-Müllerian hormone (AMH) secreted by Sertoli cells in the culture media was determined using an ELISA kit (Anshlab, Germany) according to manufacturer’s instructions. 

### RNA isolation, cDNA synthesis and reverse transcription polymerase chain reaction

Immediately after completion of cultivation, samples were transferred to sterile cryovials. To protect integrity of the RNA pool in cells, RNA later (Sigma, UK) was added to each sample. Cryovials were then immediately transferred to the -80˚C freezer. Total RNA from cultured Sertoli cells were then extracted by using the RNX kit standard (Cinnagen, Iran) according to the manufacturer’s instructions. The extracted RNA was treated with DNaseI (Fermentas, Germany) to remove genomic DNA contamination. All reverse transcription reagents were purchased from Cinnagen, Iran. cDNA was synthesized using oligo dT primers and the Superscript II reverse transcriptase system (Fermentas, Germany). 

Negative RT control was prepared by excluding the RT enzyme (minus reverse transcriptase control). The reverse transcription-polymerase chain reaction (RT-PCR) solution contained 1 μl cDNA, 22 μl Platinum blue PCR super mix (Invitrogen, UK) and 1 μl forward and the 1 μl reverse primers for *TLR2* and *TLR3* (Methabion, Germany) (Table 1). The PCR amplification comprise 40 cycles under the following conditions; initial heat at 95˚C to 60˚C for 30 seconds and final annealing at 72˚C for 30 seconds. All experiments included RT controls as well as negative controls (no cDNA).Water nuclease free controls were included to ensure lack of DNA contamination (negative control) and the endometrial samples were also used as positive controls. We used endometrial samples as a positive control because the expression of *TLR2* and *TLR3* in these cells have been shown in other previous studies (21). Furthermore, *β-actin* and glyceraldehyde-3- phosphate dehydrogenase (*GAPDH*) housekeeping genes were used as internal controls. After PCR, all samples were electrophoresed on 1.7% agarose gel (Sigma, USA) to confirm PCR amplification. 

**Table 1:** Sequence of toll like receptors (*TLR*) primers used in this study


Gene	Sequence primer (5ˊ-3ˊ)	Annealing temperature (˚C)

*TLR2*	F: TCGGAGTTCTCCCAGTTCTCT	59
	R: TCCAGTGCTTCAACCCACAA	
*TLR3*	F: GTATTGCCTGGTTTGTTAATT	59
	R: AAGAGTTCAAAGGGGGCACT	
*β-actin*	F: CAAGATCATTGCTCCTCCTG	60
	R: ATCCACATCTGCTGGAAGG	
*GAPDH*	F: CTCATTTCCTGGTATGACAACGA	60
	R: CTTCCTCTTGTCCTCTTGCT	


### Quantitative real-time polymerase chain reaction

We compared the pattern of expression of *TLR2* and *TLR3* transcripts in fibroblasts with Sertoli cells by using quantitative real-time PCR (QPCR). Ten microliter of SYBR Green Jump Start Taq Ready Mix (Sigma, USA) along with 6 µl of water and 1 µl of each primer (5 pmol) was added to 2 µl cDNA (100 ng total RNA per 25 μl of reaction mixture/μl) for each sample. The cycling conditions were 50 cycles of 95˚Cfor 15 seconds, annealing temperature of each primer (Table 1) for 30 seconds and 72˚C for 30 seconds. Data were analysed on an Applied Biosystems SDS 7000 (Applied bio system, USA). All experiments included negative controls (no cDNA). 

### Western blot analysis

Sertoli cells were lysed with lysis buffer (Qproteome mammalian protein prep kit, Qiagen, USA). Protein concentration was determined by using the Bradford protein assay (Bio-rad, USA). Equal amounts of proteins were separated by sodium dodecyl sulfate- polyacrylamide gel electrophoresis (SDS- PAGE) and subsequently electro-transferred onto polyvinyl difluoride membranes (Thermo, USA). After blocking with 2% non-fat milk in Tris-buffered saline (Sigma, USA) for 1 hour, the membranes were incubated overnight at 4˚C with TLR2 and TLR3 primary antibodies (CST, USA) at a dilution of 1:1,000. After washing twice with Tris-buffered saline containing 0.1% Tween 20, the membranes were incubated with the anti-rabbit IgG peroxidase-conjugated secondary antibodies (Sigma, USA) at a dilution of 1:100,000 at room temperature for 1 hour. Antigen-antibody complexes were visualized using an enhanced chemiluminescence detection kit (GE, USA). 

### Statistical analysis

Statistical analyses were based on ANOVA test. Data were presented as mean ± SEM. Values of P<0.05 were considered as statistically significant. All analyses were implemented in SPSS version 16. 

## Results

### Morphological analysis

**Fig.1:**
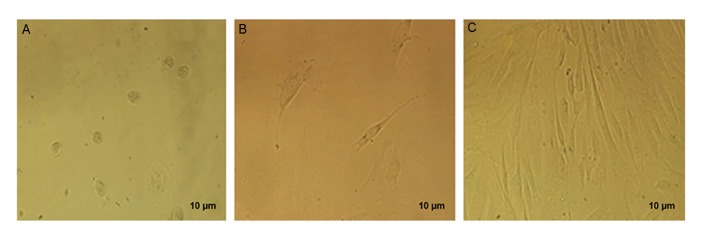
Sertoli cell cultivation in culture media in different time intervals. A. One day, B. Two days, and C. Two weeks.

The morphology of the Sertoli cells was investigated with light microscopy. These cells are granular and lumpy, and irregular margins due to lipid droplets were apparent. These cells, after isolation, lost their cytoplasmic processes and were observed with an irregular shape. In the first week of cultivation, they formed an elongated and flattened appendage, trying to make a connection with adjacent cells. After cell division, these cells made a cell layer at the bottom of the petri dish (Fig.1). 

### Alkaline phosphatase activity 

**Fig.2:**
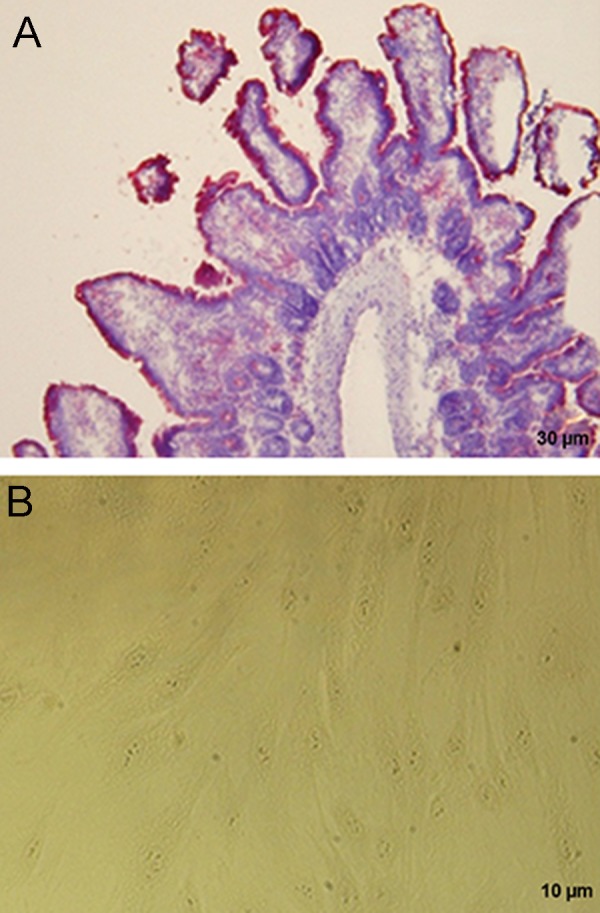
Comparison alkaline phosphatase activity in Sertoli cells. A. Mouse intestinal brush border of villous enterocytes as a control positive and B. Sertoli cells with no alkaline phosphatase activity.

To determine alkaline phosphatase activity, first, as a control positive, mouse intestinal tissue was analyzed for alkaline phosphatase activity. Investigation of mouse intestinal tissue showed that intestinal brush borders of villous enterocytes (purple color) are the location for alkaline phosphatase activity. The monolayer of Sertoli cells did not show alkaline phosphatase activity, confirming purity of isolated Sertoli cells (Fig .2). 

### Vimentin expression in Sertoli cells

**Fig.3:**
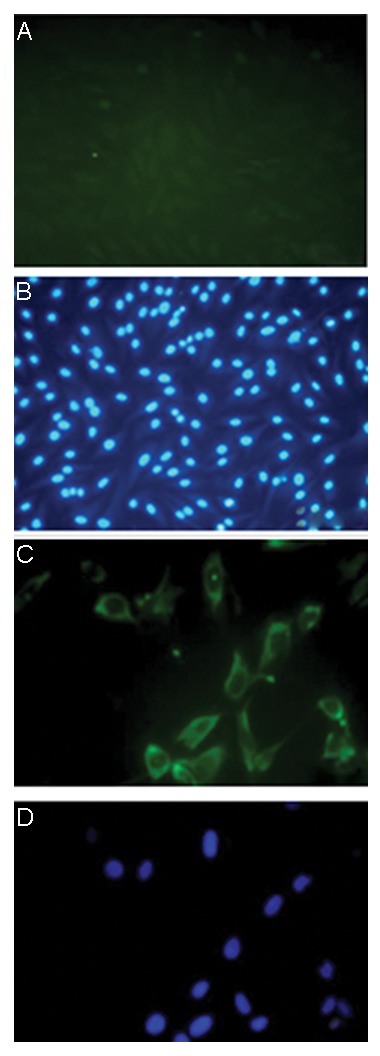
Confirmation of the nature of Sertoli cells obtained from human testis with vimentin specific markers. A, B. Fibroblasts acquired by FACS which are vimentin negative, C, and D. Sertoli cells which are vimentin positive.

The immunohistochemical staining of the isolated Sertoli cells showed that vimentin was expressed in the cytoplasm around the nucleus of nourishing cells, while fibroblast cells did not show any expression (Fig.3). 

### Presence of follicle-stimulating hormone receptors on the surface of Sertoli cells 

**Fig.4:**
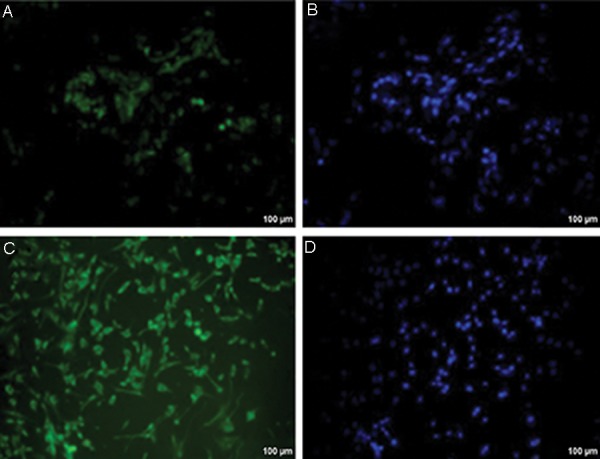
Confirmation of the nature of Sertoli cells isolated from seminiferous tubules with follicle-stimulating hormone (FSH) receptor. A, B. FSH receptor in human testicular tissue as a positive control, C, and D. FSH receptor in cultured Sertoli cells.

Immunohistochemical staining of isolated Sertoli cells showed that FSHr were expressed on the surface of the Sertoli cells and these cells were thus FSHr positive. FSHr expression on human testicular tissue was also confirmed as a positive control (Fig.4). 

### Purification of Sertoli cells with lectin FACS

**Fig.5:**
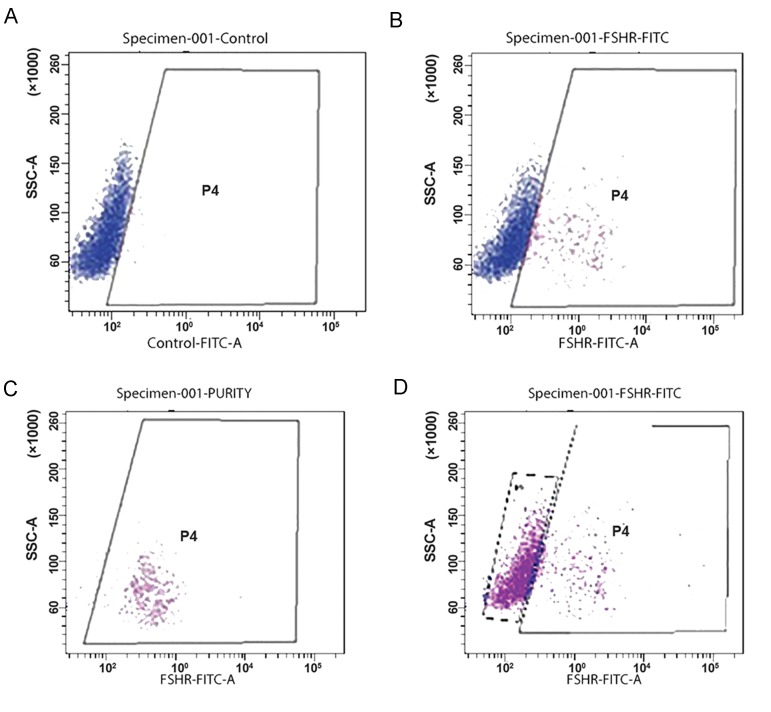
Expression of follicle-stimulating hormone receptor (FSHr) before and after isolation via FACS. A. Before sorting, B. P4 population with 25% FSHr expression, C. After sorting with 97% purification, and D. The remaining cells do not have FSHr with 75% purification.

Sertoli cells and mesenchymal cells, which are mainly fibroblasts, were isolated by FACS sorter. FSH antibody was added to the cells according to the instructions (Fig.5A). The cells that had FSHr were attached to the antibodies (p4) (Fig.5B). Sertoli cells were next purified (97%) and sorted (Fig.5C). The remaining cells (p5) did not have FSHr and were sorted with a purity of 75% (Fig.5D). These cells were cultured as Sertoli cells culture protocol. 

### Expression analysis of TLR2 and TLR3 in Sertoli cells

**Fig.6:**
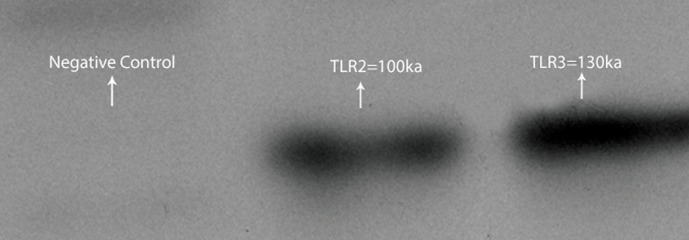
Bands obtained by western blot analysis of Toll like receptor 2 (TLR2) and TLR3.

Western blot analysis showed that the level of TLR3 protein expression was higher than TLR2 in Sertoli cells of human testicular tissue (Fig.6). 

### Expression level of *TLR2* and *TLR3* in human Sertoli cells

**Fig.7:**
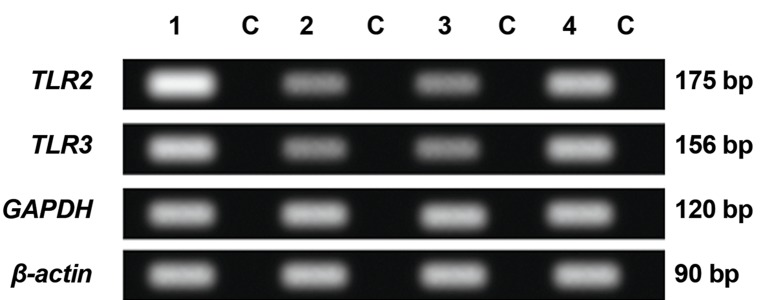
Results of real time polymerase chain reaction (RT-PCR) for *TLR2* and *TLR3* observed in 4 groups. 1; Fibroblast with Sertoli cells, 2; Sertoli cells, 3; Fibroblast cells, 4; Tissue in human endometrium (positive control) due to actin gene expression, and C; No cDNA negative control.

**Fig.8:**
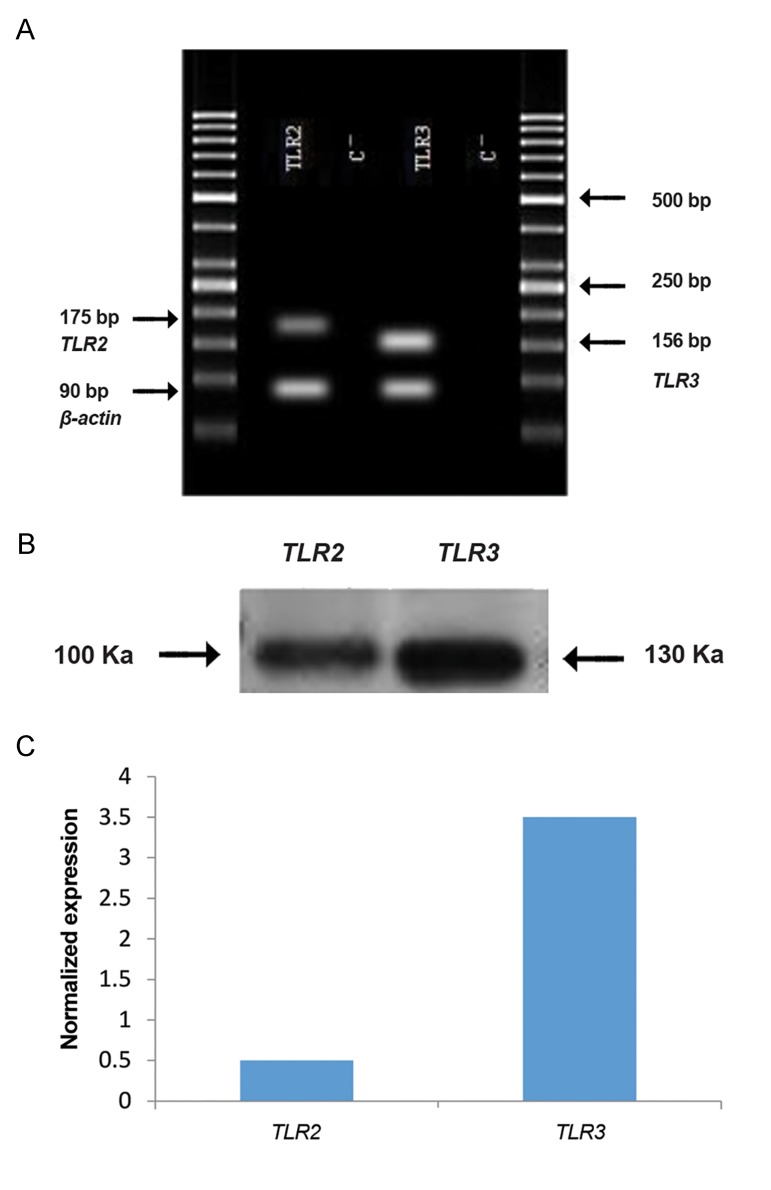
Expression of Toll like receptor 2 (*TLR2*) and *TLR3* at the transcript and protein levels in human Sertoli cells. A. Reverse transcription-polymerase chain reaction (RT-PCR), B. Western blot, and C. Quantitative real-time PCR (QPCR).

RT-PCR showed the presence of *TLR2* and *TLR3* transcripts in 4 groups including fibroblasts with Sertoli cells, fibroblast only, Sertoli cell only and human endometrium as a positive control (Fig.7). The expressions of *TLR3* in Sertoli cells were higher than *TLR2* (P<0.05) (Fig.8). 

**Fig.9:**
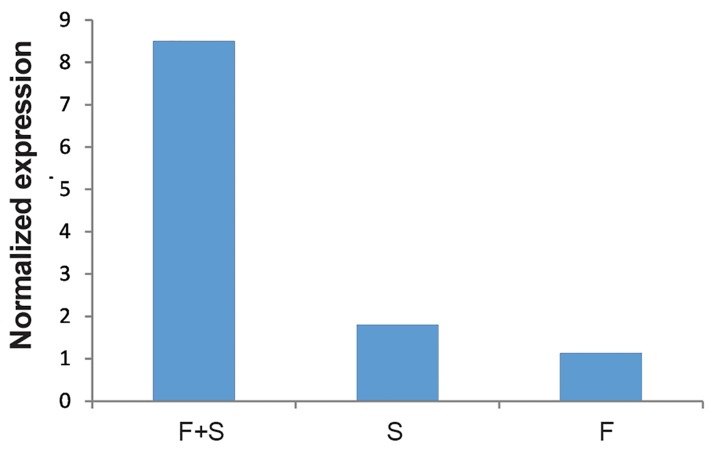
Comparison of the Toll like receptor 2 (*TLR2*) in Sertoli cells with fibroblasts (F+S), Sertoli cells (S) and fibroblast cells (F).

**Fig.10:**
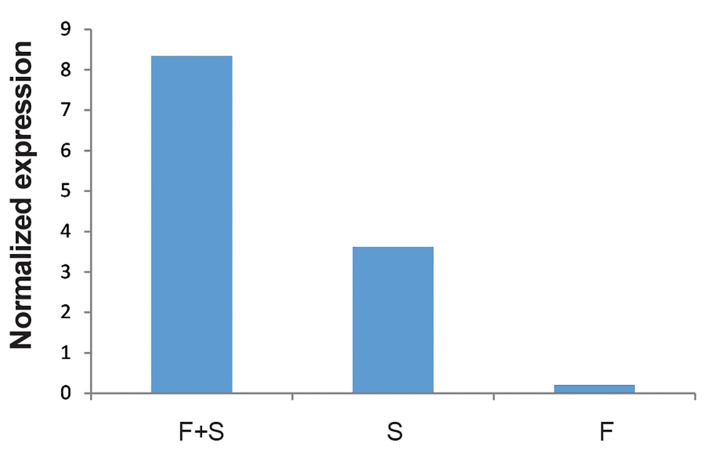
Comparison of the Toll like receptor 3 (*TLR3*) in Sertoli cells with fibroblasts (F+S), Sertoli cells (S) and fibroblast cells (F).

Expression of *TLR2* in fibroblast cells with Sertoli cells was also significantly higher than either Sertoli cell only or fibroblast only groups (P<0.05). Expression of *TLR2* in human Sertoli cells was also significantly higher than in fibroblasts (P<0.05) (Fig.9). The same patterns as for *TLR2* were also observed for *TLR3* (Fig .10). 

## Discussion

*TLR2* and *TLR3* transcript expression in Sertoli cells obtained from testicular sperm extraction (TESE) samples were studied. In the first step, Sertoli cells were isolated from testes and then grown and amplified, followed by a two-stage enzymatic digestion. Next sertoli cells were isolated and cultivated by using two complementary methods, namely DSA lectin, as the initial isolation, and FACS. In adult testis compared with immature specimens, taking these steps are much more difficult because matured testicles have different types of germ cells. Therefore, after the process of isolation, screening, and cultivation, this cell type should be confirmed with irregular margins, granular appearance and observation of a cellular monolayer *in vitro*. To confirm the nature of these cells, in addition to morphology, alkaline phosphatase activity and cell-specific markers were also tested. The existence of the cytoskeletal protein, vimentin, and FSHr studied by immunocytochemistry confirmed that DSA lectin-isolated cells were Sertoli cells.

Results of *TLR* expression in human Sertoli cells was in accordance with the findings in animal models (18, 22). ALP testing in the cells of a feeder layer showed that these cells have no ALP activity. Single cells from the testes of mice which have alkaline phosphatase activity were musclelike cells (myoid cells) surrounding vessel wall and the seminiferous tubules (23), however, the present study shows the expression of vimentin in human Sertoli cells with no ALP activity.

Previous studies have only shown the existence of *TLRs* in male and female genitalia but these receptors yet had not been studied in human Sertoli cells. Nishimura and Naito (14) reporting* TLR1-* 10 and associated factors including MYD88, TRIF, TIRAP/MAL and CD14 in prostate and testicular tissue samples. Riccioli et al. (24) examined the role of Sertoli cells in testicular innate immune responses in mice via *TLR* activity. They investigated the role of nuclear factor-κB (NF-κB) and MAPKS in the regulation of the *TLR* mediators and also demonstrated that the induction of ICAM-1 expression in Sertoli cells is caused by the stimulation of *TLR2, 5* and *6*. Palladino et al. (15) showed a strong expression of *TLR1-9* in testis, epididymis and vas deferens of mice and a weak expression of *TLR11* in testis and vas deferens. One year later, they showed the presence of *TLRs* on the sperms of mice. This study, however, showed that *TLR2* and *TLR3* are both expressed in human isolated Sertoli cells from TESE samples.

We have shown that the expression level of *TLR3* on the membrane of endosomes of Sertoli cells is higher in comparison to *TLR2*. This study was concentrated on *TLR2* and 3 for the following two reasons. First, *TLR3* is responsible for immune responses against viral infections. These infections are responsible for pathologic disorders such as orchitis, testicular cancer and male infertility. Also, *TLR2* can recognise a broad spectrum of bacterial pathogens when forming a heterodimer with *TLR1* and *TLR6* (25). Secondly, *TLR3* in identification of viral and trapped pieces in endosomes plays an important role.

The first defensive barrier in seminiferous tubules is macrophages and the second one is the Sertoli cells. The viruses are able to penetrate through both barriers. In the case of bacteria, if they are sufficiently small, e.g. with a size of virus, or are of the intracellular type, they may also pass through these barriers (16, 25). In these cases, *TLR3* on Sertoli cells can detect the entering pathogens. In the presence of infection, some cytokines such as interleukin-1B (IL-1B), IL-6 and tumor necrosis factor-alpha (TNF-α) are produced. If this is followed with uncontrolled activation of *TLR* pathways, it may lead to over-production of these cytokines and manifestation of autoimmune disorders, and consequently sterility in males. The major role in suppression of over-activation of cytokines is played by TRL3 via TAM receptors, which have an inhibitory function in regulation of immune response (26). That is possibly why *TLR3* is more abundant than *TLR2* (as observed in this study) and is constitutively expressed in Sertoli cells (3).

## Conclusion

This study implicates TLRs as a new dimension in the immune system environment in human testes. This study is however preliminary and we believe that future studies should comprehensively focus on understanding TLR biology in the reproductive system, function of sperms and Sertoli cells. Such endeavors may open the way for therapeutic applications.
